# Editorial: Advancing *Marchantia polymorpha* research: unveiling physiological, developmental, and evolutionary insights

**DOI:** 10.3389/fpls.2025.1731090

**Published:** 2025-11-17

**Authors:** Jorge Poveda

**Affiliations:** Recognised Research Group AGROBIOTECH, UIC-370 (JCyL), Department of Plant Production and Forest Resources, University Institute for Research in Sustainable Forest Management (iuFOR), University of Valladolid, Palencia, Spain

**Keywords:** *Marchantia polymorpha*, model plant, land plant evolution, plant-microorganism interaction, 3D morphological analysis, bimolecular fluorescence complementation

## 1 *Marchantia polymorpha*: a multidisciplinary model plant

Model plants are used to study various biological processes, allowing the knowledge generated to be extrapolated to plant species that are more complex to manipulate. A model plant must meet a set of characteristics, such as small size and/or manageability, short life cycle, small and/or well-annotated genome, self-compatibility, conserved nature of biological mechanisms, and ability to be genetically modified. Due to these factors, the first model plant was the angiosperm *Arabidopsis thaliana* (Brassicaceae family), which has been used to identify many plant genes and their functions. Other model plants have been established subsequently, due to their taxonomic proximity to different areas of interest in plant research, such as *Chlamydomonas reinhardatii* (Chlamydomonadaceae family), *Oryza sativa* (Poaceae family), *Zea mays* (Poaceae family), *Triticum dicoccoides* (Poaceae family), *Populus trichocarpa* (Salicaceae family) and *Picea abies* (Pinaceae family). Although these model plants are useful for studying very diverse biological processes, they do not allow us to answer questions related to how terrestrial plants have developed physiologically and morphologically, or how they have adapted to abiotic and biotic stresses. Because of these limitations, bryophytes are being considered as a possible resource for interesting model plants (Yadav et al., 2023).

Bryophytes are non-vascular, spore-forming plants that make up a monophyletic plant lineage related to all other land plants. This group of plants includes more than 16,000 different species, grouped into three different evolutionary lineages: liverworts, mosses and hornworts. Bryophytes and tracheophytes (other land plants) have a common ancestor, but colonized the land in different evolutionary events. Since bryophytes have undergone much less evolutionary diversification than their tracheophyte relatives, they represent a very interesting group of plants for the study of plant evolutionary biological processes. In this regard, *Physcomitrium patens*, *Marchantia polymorpha* and *Anthoceros agrestis* are proposed as possible model bryophyte plants (Yadav et al., 2023).

*M. polymorpha* is a liverwort (class Marchantiopsida) that has attracted considerable interest as a model plant in recent years. In addition to having important basic characteristics of a model plant, such as simple cultivation, ready access via its worldwide distribution, ease of crossing, facile genetics, efficient transformation, genome editing, and genomic resources, it is a characteristic representative for studies of evolutionary processes in land plants. For all these reasons, *M. polymorpha* is an increasingly used model plant in multidisciplinary studies that include physiological, developmental, evolutionary, abiotic and biotic interaction approaches, and for scientific and technical developments, ranging from genetic engineering to histological analysis (Bowman et al., 2022).

## 2 Model bryophyte for descriptive biological studies

The liverwort *M. polymorpha* has been known to humankind for millennia, as recorded in the herbariums of ancient Greece. In the 18th and 19th centuries, various descriptive biology studies were carried out on this bryophyte, elucidating the life cycles of this group of plants, enabling the formulation of cell theory, and providing important evidence for the discovery of the alternation of generations in land plants. Subsequently, and to the present day, *M. polymorpha* has continued to be used as a model plant in botany (Bowman, 2022).

Despite the large amount of work being carried out with *M. polymorpha in vitro* in recent years, its detailed study in natural conditions still requires further research. The work carried out by Duckett et al., and published in this Research Topic, studied the evolution of *M. polymorpha* subsp. *ruderalis* (Bischl. & Boissel. Dub) populations in two nature reserves in southern England (Thursley Common and Chobham Common) over a period of three years, following severe fires in 2020. The initial establishment of the *M. polymorpha* population was from airborne spores, not from previously existing spore banks. However, from that initial moment onwards, propagation is mainly by gemmae, whose germination has fewer requirements (Duckett et al.). In addition, the study describes in detail how the different reproductive cycles develop and how the established populations vary over the three years following the fire.

## 3 Model plant for physiological and developmental studies

The field of study that has seen the most development with *M. polymorpha* is that of physiological and developmental processes. In recent years, key genes in the bryophyte development process have been identified and described, which are functionally well conserved in land plants. For this reason, *M. polymorpha* is proposed as an excellent model plant for studying the conserved and diversified mechanisms underlying land plant development (Kohchi et al., 2021).

Recent studies, published in this Research Topic, have focused on identifying key genes in important plant physiological processes. In this regard, the *MpSPI* (*M. polymorpha SPIRRIG*) gene has been described as fundamental in cell morphogenesis and salt resistance regulation in *M. polymorpha*. In addition, *MpSPI* regulates genes in *M. polymorpha* that are orthologs of *A. thaliana* genes regulated by its *AtSPI* gene (Koebke et al.). Using a similar approach, the role of the *MpSGF10B* (*M. polymorpha SERINE AND ARGININE RICH SPLICING FACTOR 10*) gene in the reproductive development of *M. polymorpha* has also been identified. The expression pattern of this gene is closely related to that of other genes related to cell cycle and development (Kobayashi et al.). Other genes, such as *MpD27–1*, *MpD27–2* (*M. polymorpha DWARF27–1* and *2*) and *MpCCD7* (*M. polymorpha CAROTENOID CLEAVAGE DIOXYGENASE 7*), have been clearly identified as key players in controlling bud release, germination, and growth in response to variable light conditions. Interestingly, these genes are directly involved in the biosynthesis of strigolactones, a class of carotenoid-derived hormones that play a crucial role in flowering plants and interaction with symbiotic arbuscular mycorrhizal fungi (Jibran et al.).

Not only have physiological and developmental studies been conducted with *M. polymorpha* using gene knock-out mutants, but other approaches, such as omics, have also been developed in recent years. In a study conducted by Kolkas et al. (2022), the proteome of the cell wall of *M. polymorpha* was studied. This study has allowed the identification of molecular specificities in bryophytes that differ from flowering plants, such as the presence of mannans of phenolics in cell walls (Kolkas et al., 2022).

## 4 Model plant for abiotic and biotic interactions

*M. polymorpha* has also been used as a model plant in plant interactions with abiotic and biotic stresses, from an evolutionary perspective of all land plants (Beaulieu et al., 2025). Some of the most studied aspects include high temperatures (Marchetti et al., 2021), wounding (Beraldo and Alboresi, 2024) and interactions with microorganisms (Poveda, 2020).

Through its interaction with microorganisms (both beneficial and pathogenic), *M. polymorpha* is a valuable source of knowledge, as it provides information on new microbial species and bioactive compounds. Work in this area has focused on the antimicrobial metabolites produced by *M. polymorpha*, the identification and characterization of epiphytic, endophytic and pathogenic microorganisms, molecular studies of the direct interaction between *M. polymorpha* and microorganisms, and the transformation of plants using bacterial vectors (Poveda). Therefore, in recent years, significant advances have been made in understanding the interactions between *M. polymorpha* and microorganisms, both from an evolutionary and applied perspective, as discussed in the recent review by Poveda included in this Research Topic.

## 5 Model organism for the development of technical methods

In addition to generating new basic knowledge, *M. polymorpha* can be used as a model plant for the development of new tools and methodologies for experimentation and analysis. This includes the establishment of *in vitro* cultures, controlled crosses (Ishizaki et al., 2016), genetic transformation (Ishizaki et al., 2016; Sauret-Gueto et al., 2020), and both histological and cytological studies (Wang et al., 2023).

Precise three-dimensional morphological analysis is an essential tool in many biological studies, although it is still under development. In the work carried out by Furuya et al., and published in this Research Topic, *M. polymorpha* and a mutant with an abnormal 3D shape are morphologically analyzed using a new approach that combines a 3D imaging technique using micro-computed tomography and a mathematical image-processing method to describe 3D morphological features. The system made it possible to distinguish the wild type from a mutant with different morphological features, opening the door to its application in different tissues or bodies with irregular 3D morphology (Furuya et al.).

Another interesting methodological development using *M. polymorpha* as a model is a set of tools for the rapid and easy visualization of marker proteins, protein-protein interactions, and cell morphology. The work carried out by Westermann et al., and published in this Research Topic, proposes the rapid and reliable transient biolistic transformation of epidermal cells from the *M. polymorpha* thallus using fluorescent protein fusions to label a variety of subcellular compartments. In addition, the functionality of bimolecular fluorescence complementation for imaging living cells in *M. polymorpha* is confirmed, applicable for visualizing cell boundaries or cell structures, for complementing or supporting protein localizations, and for understanding how results obtained through transient transformations can be integrated into cell architecture and dynamics (Westermann et al.).

In conclusion, *M. polymorpha* has emerged as a powerful multidisciplinary model plant that bridges fundamental research in evolution, physiology and plant–microbe interactions with the development of innovative experimental methodologies. Its unique combination of ancestral traits, genetic tractability and ecological relevance provides an essential framework for understanding the evolution and diversification of land plants. This Research Topic encompasses different studies on the aspects mentioned above, representing a significant advance in basic and applied knowledge of *M. polymorpha* as a model plant.
